# Prevalence of Transfusion-Transmissible Infections Among Voluntary Blood Donors in a Tertiary Care Hospital

**DOI:** 10.7759/cureus.70469

**Published:** 2024-09-29

**Authors:** Suchita Deshmukh, Yogita Rathod, Shivani Thakore, Shivshankar Jadhav

**Affiliations:** 1 Pathology, D. Y. Patil Medical College Kolhapur, Kolhapur, IND; 2 Orthopaedic Surgery, Jawaharlal Nehru Medical College, Datta Meghe Institute of Higher Education and Research, Wardha, IND

**Keywords:** blood donors, hepatitis b, hiv, seroprevalence, syphilis, transfusion-transmissible infections

## Abstract

Background

Transfusion-transmissible infections (TTIs) pose a significant risk to blood transfusion safety, especially in low-resource settings. TTIs include infections such as HIV, hepatitis B virus (HBV), hepatitis C virus (HCV), syphilis, and malaria. Over four years, this study assesses the seroprevalence of TTIs among voluntary blood donors at a tertiary care center.

Materials and methods

This retrospective observational study was conducted at the blood bank of a tertiary care center from June 2019 to December 2022. A total of 4639 voluntary blood donors were screened for TTIs, including HIV, HBV, HCV, syphilis, and malaria, using third-generation enzyme-linked immunosorbent assay (ELISA) kits and rapid diagnostic tests. The data were analyzed to evaluate the seroprevalence of each infection and its trends over the study period.

Results

The overall prevalence of TTIs among the 4639 donors was 68 (1.46%). The highest seroprevalence was observed for HBV, with 33 (0.71%), followed by syphilis with 22 (0.47%), HIV with nine (0.19%), and HCV with four (0.08%). No cases of malaria were detected. The prevalence of TTIs was highest in 2021, with the seroprevalence of HBV peaking at 15 (1.14%). Male donors accounted for 4412 (98%) of all donations, and the prevalence of TTIs was significantly higher among males than females.

Conclusion

The study highlights the importance of continuous surveillance and screening for TTIs among blood donors to ensure transfusion safety. The higher seroprevalence of HBV underscores the need for effective vaccination programs, and the disparity in gender distribution calls for strategies to encourage female blood donations. TTIs remain a public health concern, necessitating improved donor screening and public awareness.

## Introduction

Blood transfusion is a fundamental component of modern medical practice, providing life-saving treatment in a wide range of conditions, such as severe trauma, major surgical procedures, and hematological disorders [1]. However, blood transfusions carry inherent risks, including transmitting infectious agents known as transfusion-transmissible infections (TTIs). These infections, which include HIV, hepatitis B virus (HBV), hepatitis C virus (HCV), syphilis, and malaria, pose significant risks to recipients if not properly screened during the donation process [2]. TTIs can lead to chronic health conditions or life-threatening diseases, making the safety of blood transfusion services a critical public health priority worldwide.

Globally, the World Health Organization (WHO) mandates that all donated blood should be systematically screened for these infections to ensure a safe and adequate blood supply (1). Effective screening strategies have greatly reduced the incidence of TTIs in high-income countries [3]. However, in low- and middle-income countries, including India, the prevalence of these infections remains a significant challenge. Several factors contribute to this, including the high burden of infectious diseases, inadequate screening infrastructure, and reliance on replacement donors as opposed to voluntary, non-remunerated donors [4].

With its large population and considerable healthcare needs, India faces particular challenges in maintaining blood safety. The seroprevalence of TTIs among blood donors in India varies significantly across different regions [5]. In certain rural areas, where access to advanced screening technologies may be limited, the risk of transmitting TTIs through blood transfusions is particularly concerning. Previous studies have reported a wide range of TTI prevalence in Indian blood banks. For instance, Pallavi et al. found an overall TTI prevalence of 1.35% among voluntary donors in a university hospital blood bank, with HBV being the most commonly detected infection [4]. This finding aligns with broader trends across India, where HBV is frequently the most prevalent TTI due to the virus's high transmission rate through blood and blood products [6].

In addition to HBV, HIV and HCV also represent serious risks in transfusion settings. While the prevalence of HIV has declined in recent years due to improved screening technologies, it remains a public health concern due to the long-term impact of HIV infection. The transmission of HCV, another significant blood-borne pathogen, can lead to chronic liver disease and liver cancer. A study conducted in coastal Karnataka by Singh et al. found varying TTI rates, with HIV, HBV, and HCV being the most common infections detected among donors [7]. Similarly, a study conducted in the Andaman and Nicobar Islands showed a TTI seroprevalence of 1.44%, highlighting the importance of regional monitoring and control efforts [8]. Syphilis, although less commonly reported, remains a transfusion risk due to its ability to persist in blood products. Malaria, while rare in transfusions, poses a risk in endemic regions, especially when blood donors come from areas with high malaria transmission rates. According to Mohammed et al., the prevalence of TTIs can fluctuate depending on geographic, social, and environmental factors [9].

Given the significant health risks posed by TTIs, regular monitoring of their prevalence among blood donors is crucial. Blood donors represent a healthy population that serves as a surveillance cohort for detecting emerging trends in infectious diseases. Monitoring TTI trends can provide insights into the epidemiology of these infections and help guide public health strategies to reduce their transmission [10]. In India, efforts have been made to improve donor screening, but the high burden of infections and regional disparities highlight the need for continuous vigilance. Over four years, this study evaluates the seroprevalence of TTIs among voluntary blood donors at a tertiary care center in India. The data generated from this research will help assess the safety of the blood supply and guide strategies for improving blood donation services and reducing the transmission of TTIs.

## Materials and methods

Study design and setting

This retrospective observational study was conducted at the blood bank of a tertiary care center. The study period spanned from June 2019 to December 2022, during which data from all voluntary blood donors were reviewed and analyzed for the prevalence of TTIs.

Study population

A total of 4639 voluntary blood donors who met the WHO's eligibility criteria for blood donation were included in the study. Donors were between 18 and 60 years old, had a minimum hemoglobin level of 12.5 g/dL, and were deemed healthy based on a thorough medical history and physical examination. Both male and female donors were included; however, the donor pool was predominantly male.

Blood collection and handling

Blood collection from eligible donors was carried out under strict aseptic conditions to ensure donor safety and blood samples' integrity. Each donor was carefully assessed for eligibility based on predefined criteria, including their health status, medical history, and hemoglobin levels. Once deemed eligible, the donors were seated comfortably, and their blood was drawn using a sterile, single-use needle. A standard unit of approximately 450 mL of whole blood was collected from each donor. Immediately after collection, the blood was labeled and stored in sterile bags containing an anticoagulant solution to prevent clotting. The collected blood was then processed in the blood bank. Whole blood was separated into its main components: red blood cells (RBCs), plasma, and platelets. The separation process was achieved through centrifugation, ensuring that each component could be used effectively for different clinical purposes based on the needs of the patients. In addition to the main unit of blood, smaller samples were drawn into test tubes for laboratory screening. These samples were crucial for serological testing to detect TTIs. The blood collected in tubes was transported to the laboratory for immediate analysis. When necessary, care was taken to maintain the chain of custody and the cold chain to preserve the samples' integrity until testing was performed.

Screening for TTIs

To ensure the safety of the blood supply and minimize the risk of TTIs, all blood samples underwent a thorough screening process. Each sample was tested for major infections that can be transmitted through blood transfusions, including HIV, HBV, HCV, syphilis, and malaria. Various diagnostic methods were employed to detect these infections. Third-generation enzyme-linked immunosorbent assay (ELISA) kits were used to detect HIV, HBV, and HCV [11]. ELISA is a widely accepted and highly sensitive method for identifying the presence of specific antibodies or antigens related to these viruses in the donor's blood. The ELISA method can detect HIV antibodies, HBV surface antigen (HBsAg), and HCV antibodies, allowing for the early identification of infections. Each positive result was followed up with confirmatory testing to ensure the accuracy of the diagnosis and to prevent the use of infected blood for transfusion. For syphilis, the venereal disease research laboratory (VDRL) test was utilized [12]. This test is based on detecting antibodies produced in response to the *Treponema pallidum* bacterium, which causes syphilis. The VDRL test is sensitive and cost-effective, making it a practical choice for screening blood donors. Positive results were confirmed with more specific tests, if necessary, to rule out false positives.

Screening for malaria was conducted using rapid diagnostic tests (RDTs) [13]. These tests are designed to detect malaria parasites in the blood by identifying antigens derived from the parasite's presence. Although malaria transmission via blood transfusion is rare, the risk is significant in endemic regions, making it essential to include this screening in the blood safety protocol. The combined use of these advanced screening technologies ensured that only safe and infection-free blood was available for transfusion. Any blood samples that tested positive for TTIs were discarded following strict safety guidelines, and donors who tested positive were counseled and referred for appropriate medical care. This rigorous screening process was essential in maintaining a safe blood supply and protecting recipients from the potential transmission of serious infections.

Data collection

The data collection process for this study involved a comprehensive review of donor records maintained at the blood bank of the tertiary care center. Information was systematically gathered from electronic databases, ensuring accuracy and completeness. The key donor data recorded included demographic information, such as age and gender, along with each donor's donation history, which detailed the frequency and timing of their blood donations over the study period. In addition to demographic data, the TTI screening results were meticulously documented for every donor. Each donor's blood sample was screened for HIV, HBV, HCV, syphilis, and malaria, with results indicating whether the donor tested positive or negative for any of these infections. These screening results were essential for calculating the seroprevalence of TTIs among the donor population. The data collection also involved extracting information on the number of donations made each year, allowing for a year-wise analysis of donation trends. This included capturing how external factors, such as the COVID-19 pandemic, influenced donation rates over time. All collected data were recorded in a standardized format to ensure uniformity and easy analysis. Once all the relevant data were gathered, they were organized into tables, with the total number of donations and the incidence of TTIs being calculated for each year of the study. These tabulated data were subsequently used to assess the seroprevalence of each infection over the four-year study period and to identify trends, patterns, and potential areas of concern regarding blood safety. This systematic approach ensured the collected data were reliable, consistent, and suitable for meaningful statistical analysis.

Statistical analysis

The data were analyzed using IBM SPSS Statistics for Windows, Version 23 (Released 2015; IBM Corp., Armonk, New York) and Microsoft Excel 2019 (Microsoft Corporation, Redmond, Washington). Descriptive statistics were employed to summarize the demographic characteristics of the blood donors and the prevalence of TTIs. The seroprevalence rates for each infection (HIV, HBV, HCV, syphilis, and malaria) were calculated as percentages of the total donor population. Year-wise and gender-wise comparisons were made to examine trends in donation and infection rates over the study period. A chi-square test was applied to assess the statistical significance of differences in TTI prevalence between male and female donors. A p-value of less than 0.05 was considered statistically significant. Additionally, linear regression analysis was conducted to evaluate trends in TTI prevalence over time, identifying any significant increases or decreases in infection rates during the study period.

Ethical considerations

The study was conducted according to the ethical standards of the institutional review board from D. Y. Patil Medical College Kolhapur, and all donor data were anonymized to maintain confidentiality. Informed consent was obtained from all donors at the time of donation in compliance with ethical guidelines.

## Results

A total of 4639 blood donors were screened for TTIs between June 2019 and December 2022. The overall prevalence of TTIs during the study period was found to be 1.46%, with varying rates of HIV, HBV, HCV, syphilis, and malaria. Among the donors, 98% were males and 2% were females. A year-wise breakdown showed that the number of donations was impacted by the COVID-19 pandemic, particularly in 2019 and 2020, but there was a notable recovery in 2021 and 2022 (Table 1).

**Table 1:** Year-wise and gender-wise distribution of voluntary blood donors (2019–2022)

Year	Male Donors (%)	Female Donors (%)	Total Donations
June-Dec 2019	319 (94%)	20 (5.9%)	339
2020 (Jan-Dec)	919 (97.3%)	25 (2.65%)	944
2021 (Jan-Dec)	1253 (95.43%)	60 (4.5%)	1313
2022 (Jan-Dec)	1921 (94.02%)	122 (5.97%)	2043
Total	4412 (95.1%)	227 (4.9%)	4639

The prevalence of TTIs was highest in 2021, particularly for HIV and HBV, with a smaller number of infections detected in 2020 and 2022. No malaria cases were identified during the study period (Table 2)

**Table 2:** Year-wise distribution of seroprevalence of transfusion-transmissible infections (2019–2022) HIV: human immunodeficiency virus; HBV: hepatitis B virus; HCV: hepatitis C virus

Year	HIV	HBV	HCV	Syphilis	Malaria
2019 (June-Dec)	0 (0%)	1 (0.29%)	0 (0%)	1 (0.29%)	0 (0%)
2020 (Jan-Dec)	3 (0.32%)	7 (0.74%)	3 (0.32%)	6 (0.63%)	0 (0%)
2021 (Jan-Dec)	5 (0.38%)	15 (1.14%)	1 (0.08%)	7 (0.53%)	0 (0%)
2022 (Jan-Dec)	1 (0.05%)	10 (0.49%)	0 (0%)	8 (0.39%)	0 (0%)
Total	9 (0.19%)	33 (0.71%)	4 (0.08%)	22 (0.47%)	0 (0%)

**Figure 1:**
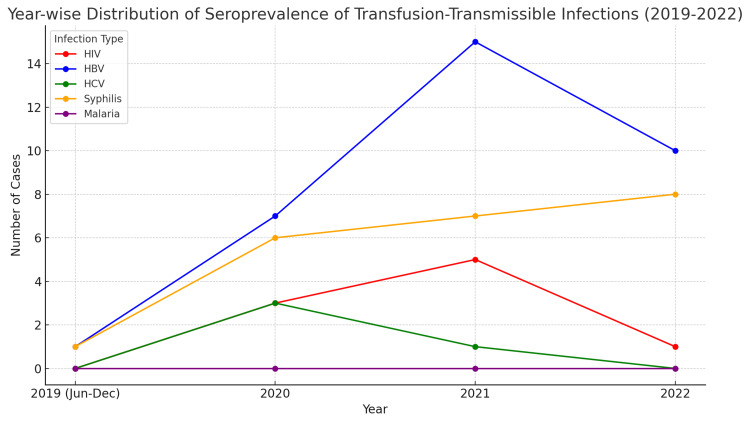
Year-wise distribution of seroprevalence of transfusion-transmissible infections (TTIs) among voluntary blood donors (2019–2022) HIV: human immunodeficiency virus; HBV: hepatitis B virus; HCV: hepatitis C virus

In terms of gender, male donors had a higher prevalence of HBV, HCV, and HIV compared to female donors, while the prevalence of syphilis was notably higher in female donors (Table 3). The year-wise distribution of seroprevalence of TTIS among voluntary blood donors (2019-2022) is shown in Figure 1.

**Table 3:** Gender-wise seroprevalence of transfusion-transmissible infections (2019–2022) HIV: human immunodeficiency virus; HBV: hepatitis B virus; HCV: hepatitis C virus

Disease	Male Seropositivity (%)	Female Seropositivity (%)	Total Seropositivity (%)
HBV	32 (0.72%)	1 (0.44%)	0.71%
HCV	4 (0.09%)	0 (0%)	0.08%
Syphilis	18 (0.40%)	4 (1.76%)	0.47%
HIV	9 (0.20%)	0 (0%)	0.19%
Malaria	0	0	0

## Discussion

Blood transfusion is a critical life-saving procedure, but it is not without risk, particularly regarding the transmission of infections. TTIs remain a significant public health concern, especially in resource-limited settings such as India, where the prevalence of infections such as HIV, HBV, HCV, and syphilis is considerable. In this study, the overall prevalence of TTIs among voluntary blood donors was found to be 1.46%, with HBV being the most prevalent infection (0.71%), followed by syphilis (0.47%), HIV (0.19%), and HCV (0.08%). The prevalence of TTIs observed in this study is consistent with previous studies conducted in India. For instance, Pallavi et al. reported a similar overall seroprevalence of 1.44% in a study conducted in South India. HBV was also the most commonly detected infection, with a prevalence of 0.87% [4]. The variation in prevalence rates across different studies can be attributed to regional differences in donor demographics, screening methodologies, and the effectiveness of public health interventions to reduce the spread of these infections.

One notable finding of this study is the higher prevalence of TTIs among male donors (98%) compared to females (2%). This gender disparity is consistent with other studies conducted in India and globally, where males are the predominant blood donors [6,14]. Cultural and social factors, including the perception that males are healthier or more eligible to donate blood, may contribute to the lower participation of female donors [15]. Encouraging female participation in blood donation programs is essential for improving the blood supply and ensuring better gender representation. HBV, with a seroprevalence of 0.71%, remains a major public health challenge in India. This rate is lower than the 1.18% reported by Karkee et al. but higher than the 0.62% found in a study conducted by Arora et al. in Southern Haryana [6,16]. The relatively high prevalence of HBV underscores the importance of vaccination programs and public awareness campaigns aimed at reducing the transmission of HBV through blood transfusions. Although a safe and effective vaccine for HBV is available, the burden of this infection remains significant, particularly in rural and low-income populations where access to vaccination is limited.

The prevalence of HIV (0.19%) in this study is comparable to findings from other Indian studies, such as Chattoraj et al., who reported a prevalence of 0.13% [17]. This relatively low prevalence reflects the success of blood screening protocols and public health initiatives aimed at reducing HIV transmission. However, the continued detection of HIV-positive donors highlights the importance of maintaining rigorous screening protocols and educating the public about the risks associated with HIV transmission through blood transfusion. Syphilis, with a prevalence of 0.47%, is a concern in this study, particularly given the higher prevalence among female donors (1.76%) compared to males (0.40%). The higher rate of syphilis among women may be linked to social and biological factors that increase their vulnerability to sexually transmitted infections (STIs) [18]. This finding emphasizes the need for targeted screening and treatment interventions for syphilis, especially in female donors, to prevent transmission through blood transfusion.

HCV had the lowest prevalence in this study (0.08%), which is in line with other studies, such as that conducted by Gupta et al., which reported a prevalence of 0.09% [19]. HCV transmission through blood transfusion is less common due to stringent screening measures, but the risk remains, particularly in regions with high HCV prevalence. Continued vigilance in screening blood donors for HCV is essential for ensuring transfusion safety. This study's absence of malaria cases is noteworthy, especially given that malaria is endemic in certain regions of India. The zero prevalence of malaria among the donors in this study may reflect the effectiveness of preventive measures, such as the use of insecticide-treated bed nets and antimalarial campaigns, as well as the geographical characteristics of the study location, which may have a lower malaria burden compared to other regions [20].

Limitations

This study was conducted at a single tertiary care center, which may limit the generalizability of the findings to other regions. Additionally, retrospective data and screening methods may not capture emerging or less common infections. The gender imbalance in donor representation could also affect the overall interpretation of the results, particularly for female donors. Finally, the impact of the COVID-19 pandemic on donor rates may have influenced the observed trends.

## Conclusions

The findings of this study underscore the ongoing risk of TTIs in voluntary blood donations, with an overall seroprevalence of 1.46%. HBV remains the most prevalent TTI, requiring strengthened vaccination efforts and enhanced screening protocols. The data also reveal that male donors overwhelmingly dominate the donor pool, highlighting the importance of encouraging greater female participation in blood donation programs. While the study detected a low prevalence of HIV and HCV, these infections continue to pose serious risks, necessitating vigilant screening and continuous monitoring to maintain blood safety. The absence of malaria cases may reflect effective preventive measures or regional epidemiological factors. These findings emphasize the critical role of regular surveillance and the need for improved public health strategies aimed at reducing the transmission of TTIs through blood transfusion. Promoting voluntary donations and ensuring stringent screening processes are essential for safeguarding the blood supply and the broader population.
